# Ultrasound Biomicroscopy Detects Peters' Anomaly and Rieger's Anomaly in Infants

**DOI:** 10.1155/2020/8346981

**Published:** 2020-03-23

**Authors:** Wen-Si Chen, Dao-Man Xiang, Lan-Xiang Hu

**Affiliations:** Department of Pediatric Ophthalmology, Guangzhou Children's Hospital & Guangzhou Women and Children's Medical Center, Guangzhou Medical University, No. 9 Jinsui Road, Guangzhou 510623, China

## Abstract

**Aim:**

Congenital corneal opacities (CCOs) are the major causes of early visual deprivation in infants. Balloon ultrasound biomicroscopy (UBM) examination is an effective method to diagnose CCO. However, whether it is suitable for children examination is still unknown.

**Methods:**

26 Peters' anomaly (PA) or Rieger's anomaly (RA) infants with congenital corneal opacities (CCO) (40 eyes) underwent UBM examinations to study their imaging features.

**Results:**

Based on the results, they were divided into UBM Dx-Type I: Descemet's membrane (DM) and endothelium have heterogenous or discontinuous echo accompanied with corneal stroma echo-enhanced or shallow anterior chamber. Type II: Type I alteration plus abnormal strand of iris extended to the border of the posterior corneal defect or iridocorneal adhesion. Type III: Type I or II combined with the abnormal hyperechoic lens, lens luxation, or keratolenticular adhesion. Type IV: echoes of the DM and the endothelium are continuous, corneal stroma echo is enhanced, and an abnormal strand of peripheral iris extends to the prominent Schwalbe line, accompanied by iris stroma or pupil heteromorphism and a shallow or flat anterior chamber.

**Conclusion:**

UBM not only could accurately evaluate the anterior segment abnormalities in CCO infants but also would be a step forward for the management of PA- and RA-associated CCO.

## 1. Introduction

Congenital corneal opacities (CCOs) are uncommon, occurring in only 2.2–3.1 per 100,000 newborns each year. CCO interferes with vision, leading to early visual deprivation and a lifetime of severe amblyopia, which becomes the major causes of early visual deprivation in infants [1, 2]. The pathogenesis of CCO can be genetic, infectious, developmental, metabolic, traumatic, glaucomatous, toxic, idiopathic, or a combination of them [3]. Anterior segment dysgenesis is one of the primary causes, among which, Peters' anomaly (PA), sclerocornea, and Rieger's anomaly (RA) may be considered as the major distinct entities of primary disorders [4–6]. PA and RA are associated with the abnormal differentiation of embryonic neural crest cells, which affect the structural development of the cornea, iris, and trabecular meshwork. Therefore, it is often accompanied by secondary glaucoma [7]. However, the early diagnosis of PA and RA with CCO is challenging [8]. The slit lamp procedure limits the early diagnosis of PA and RA due to the different opacities of the cornea as it is difficult to clearly define the lesion scope of the anterior segment structure. The oculists could not acquire enough data to analyze the anterior chamber, resulting in missed diagnosis or misdiagnosis of PA and RA during early disease stages. Indeed, few infantile patients with PA and RA could be diagnosed early with the disease, until the histological examination after penetrating the keratoplasty were performed [9]. Therefore, the discovery of a method to diagnose PA and RA early is a tricky topic for ophthalmologists.

Ultrasound biomicroscopy (UBM) is a noninvasive ultrahigh frequency ultrasound imaging system. It has been widely applied in the examination of disorders of the anterior segment of the eyeball, which provides ultra-high-frequency sound imaging to analyze the structure of the anterior segment and severe corneal opacities in living body [10]. In the current decade, most of the UBM studies are focused on glaucoma, ocular trauma, and cataracts [11–13]. Those studies are mainly for adults or older children. Some authors have studied the correlation between UBM images and histological diagnosis of CCO and found a high degree of consistency [14]. However, most of the exams were performed under anesthesia because of the non-cooperation of infants, which hindered the promotion of UBM as a routine examination in the clinical evaluation of infants with CCO. Therefore, it is uncertain whether UBM could act as a routine examination tool in infants. The clinical value of UBM as a diagnostic tool to evaluate infants with CCO is unclear. Furthermore, no studies have classified the UBM imaging features for the CCO of PA and RA to provide a diagnostic standard for imaging.

In this study, we prospectively studied the application of the high-resolution bag/balloon UBM technique as a routine examination for infant patients under sedation and surface anesthesia. This study will provide insight into the safety of UBM examination in infants, the value of UBM imaging, and the potential diagnostic capabilities of UBM in infants with PA and RA.

## 2. Materials and Methods

### 2.1. Patients

This study was approved by the Guangzhou Women and Children's Medical Center Review and Ethics Board. All CCO infantile patients were recruited from the Guangzhou Women and Children's Medical Center in Guangdong, China, between November 2012 and November 2017. Corneal opacities were the first symptom in all cases. Patients with congenital glaucoma or posterior segment diseases, like retinopathy of prematurity, persistent fetal vasculature, or retinoblastoma were excluded from this study.

### 2.2. UBM Examination

**Figure 1:**
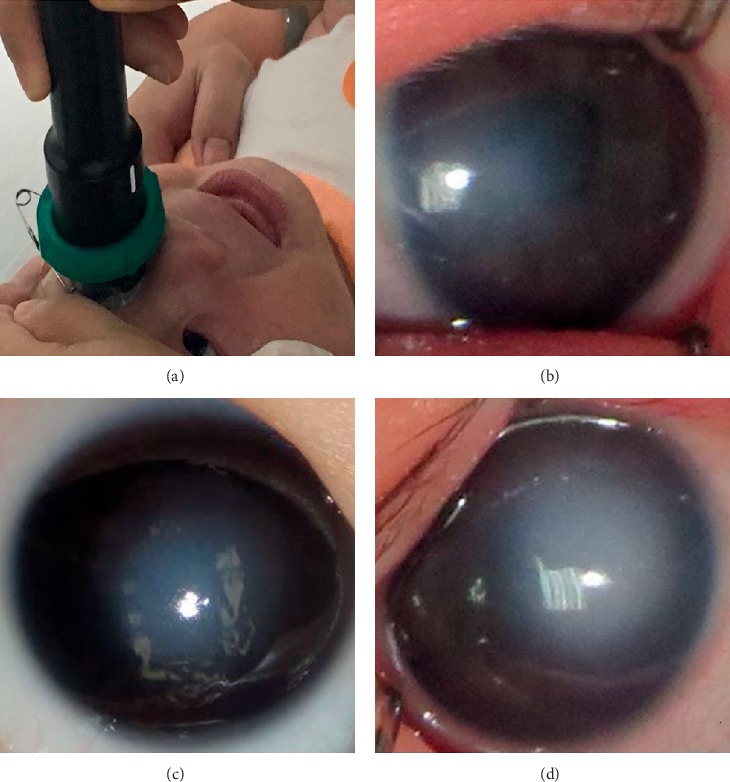
Clinical images of CCO. (a) UBM examination in awaked infants and toddlers. (b–d) The corneal opacity was divided into three clinical types. Mild: corneal haze, anterior segment structure could be easily observed (b) Medium: anterior segment structure is indistinctive (left eye of patient 26). (c) Severe: corneal leucoma, anterior segment structure could not be observed (patient 14). (d) Right eye of patient 26.

All patients received slit-lamp microscopy and high-resolution bag/balloon UBM examination using the Aviso S instrument 50 MHz (Quantel Medical, Clermont-Ferrand, France) to detect PA and RA under oral chloral hydrate sedation and tetracaine surface anesthesia in the supine position. Scans were performed by the same experienced technician, while a nurse fixed the head of patients. A sterile balloon was filled to the top of the flexible sealing collar with room temperature distilled water (Figure 1(a)). A gel was not required for the examination. The examination consisted of a minimum of four scans radial to the limbus and four scans parallel to the limbus at positions 3, 6, 9, and 12 o'clock. At least one scan axial to the estimated position of the pupil was also performed. The size, extent, and thickness of the anterior segment of the patients were captured and recorded by UBM. In addition, the relationship between the lesion and the surrounding tissues were comprehensively investigated.

### 2.3. Statistical Analysis

All data were analyzed using SPSS 23.0 (IBM, Chicago, USA).

## 3. Results

### 3.1. Demographic Characteristics

In total, 50 CCO eyes were preliminarily screened and 10 eyes were excluded due to congenital glaucoma. Finally, 40 eyes of the 26 CCO infants with PA or RA were enrolled in this study. Among them, 13 cases were male (22 eyes) and 13 patients were female (18 eyes). From these patients, 12 cases were unilateral (9 right eyes, 3 left eyes) and 14 were bilateral (28 eyes). The average age at the first visit to the hospital was 344.7 days with a range of 7 days to 3 years (Table 1).

### 3.2. Clinical Classification

According to the degree of the anterior segment structure observed through the opacity cornea, the corneal opacity was divided into three clinical types. Mild: corneal haze, anterior segment structure could be observed. Medium: anterior segment structure is indistinctive. Severe: corneal leucoma, anterior segment structure could not be observed (Figures 1(b)–1(d)). As shown in Table 1, 14 CCO patients were classified as severe (48.3%), 10 were medium (34.5%), and 5 were mild (17.2%).

### 3.3. UBM Examination and Classification

The high-resolution bag/balloon UBM examination captured clear images of the CCO patients under sedation and surface anesthesia. The pathological alterations in the anterior segment structure of all the patients with PA and RA were observed and evaluated by UBM. According to the anatomical position of the anterior segment, we distinguished the variation of the anterior segment tissue structure (cornea, anterior chamber, iris, and lens). The detailed findings of the UBM for each CCO patients are summarized in Table 2.

**Figure 2:**
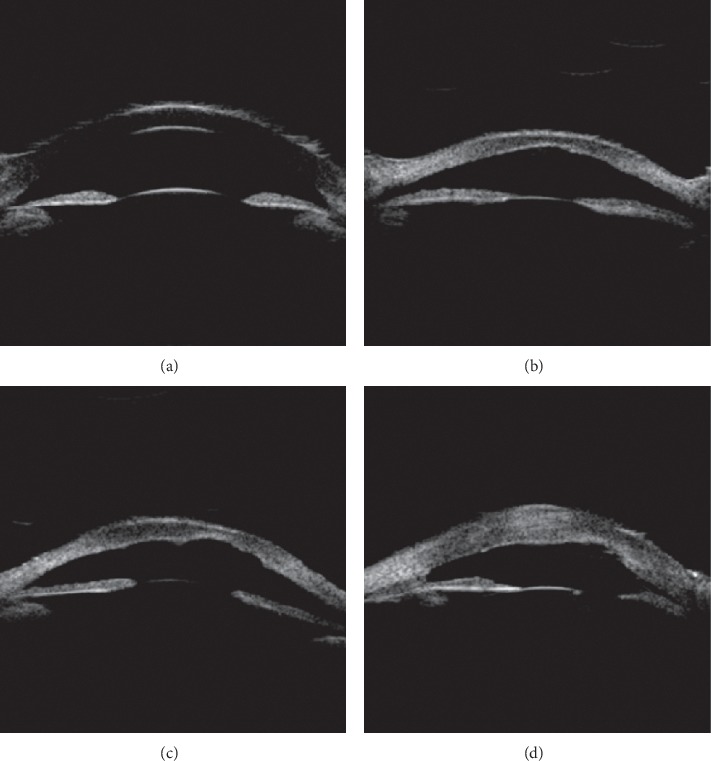
UBM images of UBM Dx-Type I (a) UBM image of the anterior segment in a healthy infant. (b–d) UBM Dx-Type I Descemet's membrane (DM) and endothelium with heterogenous or discontinuous echo (left eye and right eye of patient 8 and the left eye of patient 21, respectively). Arrows indicated the heterogenous or discontinuous echo accompanied in the DM and endothelium.

**Figure 3:**
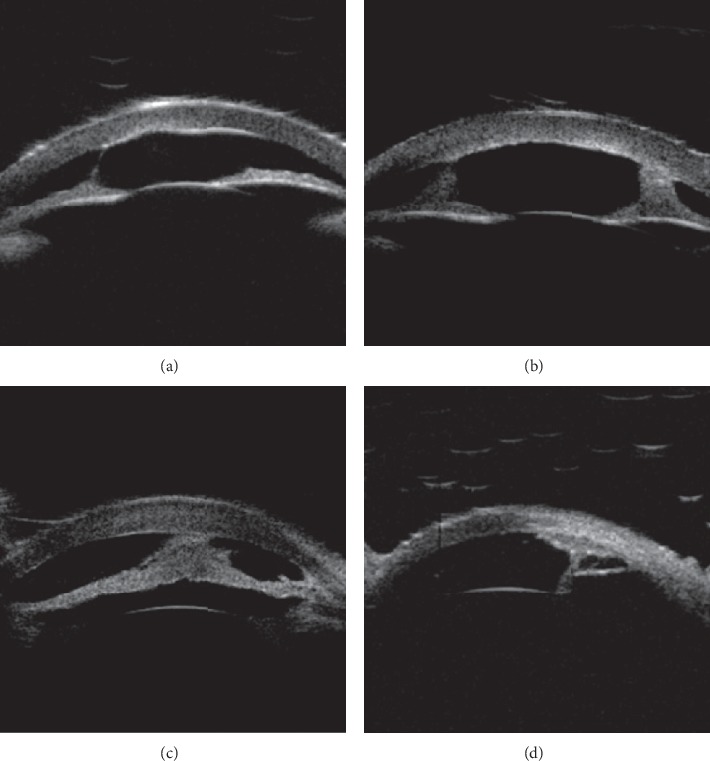
UBM images of Dx-Type II. (a) Based on the alteration of UBM Dx-Type I, we found that the abnormal filamentous iris extended to the border of posterior corneal defect (arrow in patient 18). (b) The abnormal bilateral strands of iris extended to the border of posterior corneal defect (arrow in patient 12 right eye). (c) DM and endothelium with iridocorneal adhesions (arrow in patient 12 left eye). (d) An abnormal strand of iris extended to the distention area of the DM and endothelium (arrow in patient 9).

**Figure 4:**
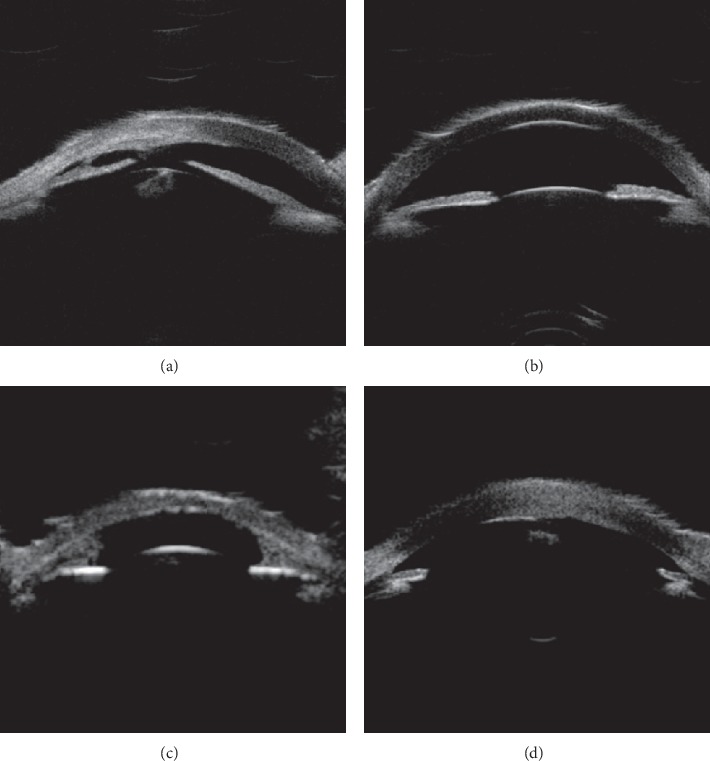
UBM images of Dx-Type III. (a) Based on the alteration of UBM Dx-Type I or II, we discovered the antedisplacement irises, enhanced lens' echo, and penetration the anterior chamber (arrow in patient 17 right eye). (b) Discontinuous echo on the anterior surface of the irises with iridocorneal adhesion, and the enhanced echo of the lens (arrow in the left eye of patient 17). (c) Adhered bilateral irises to the peripheral cornea and enhanced lens' echo penetrated into the anterior chamber (arrow in patient 4). (d) Enhanced lens' echo penetrated into the anterior chamber, near the posterior surface of the cornea (arrow in patient 14).

**Figure 5:**
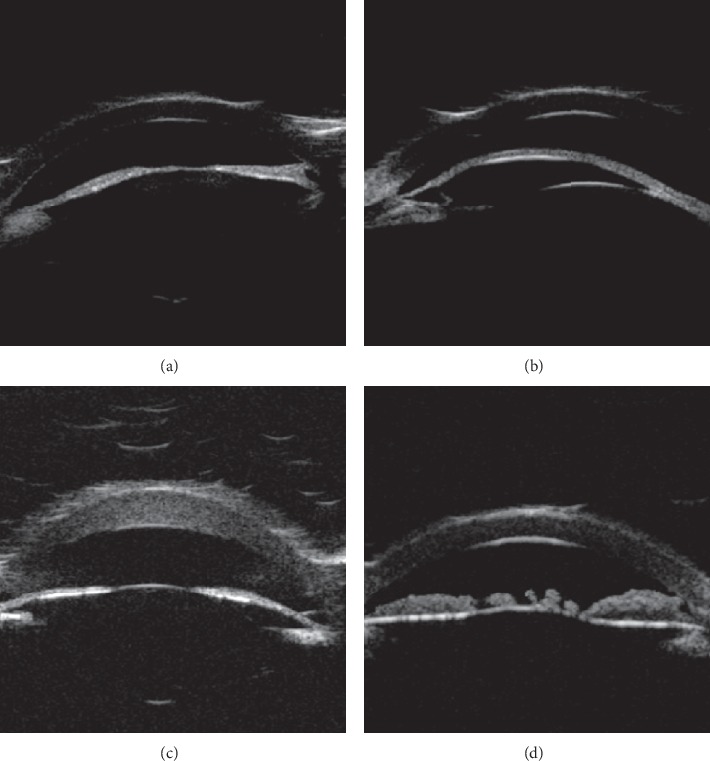
UBM images of Dx-Type IV. (a) A flat echo consistent with the echo intensity of the iris in the pupil's area, and an abnormal strand of peripheral iris extends to the protruding Schwalbe line (arrow in patient 3). (b) The iris was antedisplacement, an abnormal strand of peripheral iris extended to the protruding Schwalbe line, and the pupil disappeared (arrowed in patient 26). (c) An abnormal strand of peripheral iris extended to the protruding Schwalbe line (arrow in patient 1). (d) The iris thickened, a discontinuous echo consistent with the echo intensity of the iris in the pupil's area, and an abnormal strand of peripheral iris extends to the protruding Schwalbe line (arrow in patient 13).

Based on the alteration of the histological structure of the anterior segment and the imaging features from the UBM examination, the patients were classified into the following four types (Table 3): UBM Dx-Type I: UBM found that Descemet's membrane (DM) and endothelium had heterogenous or discontinuous echo accompanied with corneal stroma echo-enhanced or shallow anterior chamber. Six eyes (cases 8 and 19 were bilateral, and cases 11 and 21 were left eyes) were divided into this category (Figure 2). UBM Dx-Type II: the alteration of UBM Dx-Type I plus abnormal strand of iris extend to the border of posterior corneal defect or iridocorneal adhesion. Sixteen eyes (cases 2, 6, and 12 were bilateral, cases 9, 11, 15, 18, 21, 24, and 26 were right eyes, and 16, 22, and 23 were left eyes) were classified into this type (Figure 3). UBM Dx-Type III: the findings of UBM Dx-Type I or II combined with an abnormal hyperechoic lens, lens luxation, or keratolenticular adhesion. Twelve eyes (cases 5, 14, and 17 were bilateral, cases 4, 7, 10, 20, and 23 were right eyes, and case 15 was the left eye) were divided into this type (Figure 4). UBM Dx-Type IV: the echoes of the DM and the endothelium were continuous; with corneal stroma echo-enhanced, an abnormal strand of peripheral iris extends to protruding Schwalbe line, often accompanied with iris stroma or pupil heteromorphism, with shallow or flat anterior chamber. Six eyes (case 1 was bilateral, case 3 was the right eye, and cases 13, 25, and 26 were left eyes) were classified into this category (Figure 5).

## 4. Discussion

In this study, we reported the UBM findings in 40 eyes from 26 infants with PA- and RA-associated CCO. The high-resolution ball/balloon UBM technique was applied in patients with an age range of 7 days to 3 years (mean, 344.7 days). All infantile patients were successfully examined by UBM under sedation and surface anesthesia to obtain valuable images. All UBM images revealed the structural features of the cornea, anterior chamber, iris, and lens. To our knowledge, this is the most extensive study to apply the high-resolution ball/balloon UBM technique to diagnose PA- and RA-associated CCO in infants so far.

UBM examination is a noninvasive, real-time, fast, and high-resolution detection method to observe the structure of the anterior segment from the living body. The images provide sectional visibility of anatomical sections, which show the structure of the anterior segment in high resolution. It is especially suitable for the patient whose cornea is opaque to clearly display the relationship between the cornea, iris, ciliary body, chamber angle, lens, and surrounding tissue in a noninvasive manner. Thus, it is valuable for the role of the histological examination of living tissue [15, 16]. In recent years, UBM has been applied for the diagnosis of PA or RA patients in some isolated case reports. Shigeyasu et al. retrospectively studied the UBM application on CCO in adult and older children patients [5, 17–19]. In addition, Nischal have reported the UBM examination under anesthesia in 13 cases of infants with CCO. In that study, the UBM findings were found to be identical to the clinical features (in all cases) and the histological alterations (in 9 cases). Indeed, the UBM findings changed the clinical diagnosis in 5 of the cases [20].

Most ophthalmologists still use the traditional open-shell UBM technique. It is necessary to choose the appropriate eye cup according to the size of the palpebral fissure. Thus, it is only suitable for most adult patients. However, it is not ideal for the adults and infants whose palpebral fissures are small, as it may cause discomfort and non-cooperation during the examination, especially for children [21]. During the examination, the corneal epithelium could be damaged if the eyecup is positioned improperly, if the eyes move, or if the probe is exposed to the corneal epithelium. In addition, the open-shell UBM technique is limited to the examination of the posterior limbus area. This study applied the high-resolution ball/balloon UBM technique, which provides a water bag covering on the end of the UBM probe to avoid direct contact with the cornea. It is a convenient, safe, tolerable, and relatively painless method [22], which is beneficial for examining infants under sedation. Furthermore, it improves the image definition and effectively displays the surrounding area of the cornea. It can obtain a two-dimensional image of the anterior segment structure on any meridian, while also enlarging the examination range and improving the diagnostic accuracy [23].

According to the UBM finding features, the cases were classified into four types (UBM Dx-Type I to IV). Waring et al. have classified PA and RA based on the characteristics of histological and anatomical alterations [24]. Indeed, our classification referenced their method and was highly consistent with their study protocol (Table 4) but without any biopsies.

Although the UBM images of UBM Dx-Type I to III varied, they showed the heterogenous or discontinuous echo in the DM and endothelium, which histologically corresponds with the PA's alteration of specific local defects. UBM Dx-Type I only has the above image features, but its iris and lens were normal. UBM Dx-Type II accompanies with an iris anomaly, such as the abnormal strand of iris extending to the border of the posterior corneal defect or with iridocorneal adhesion. This image feature is consistent with the type of posterior corneal defect common with stromal edema and iridocorneal adhesion in PA. The images of UBM Types I andII are consistent with the PA Type I of Waring's classification. UBM Dx-Type III is on the fundamental alteration of UBM Dx-Type I or II, plus the abnormal hyperechoic lens, lens luxation, or keratolenticular adhesion. Its image feature is consistent with the type of posterior corneal defect with stromal edema, cataract formation, or keratolenticular in PA, also known as the PA type II.

The UBM image of UBM Dx-Type IV (the continuous echoes of the DM and endothelium with the abnormal strand of peripheral iris extended to the protruding Schwalbe line, often accompanying iris stroma or pupil heteromorphism) is consistent with the type of RA, including peripheral anterior adhesion that strands of iris tissue to the Schwalbe line and uplifts as a ring, iris hypoplasia, polycoria, and corectopia. In this observation of 26 cases (40 eyes), 22 eyes were divided as PA type I (55%), 12 eyes were diagnosed as PA type II (30%), and 6 eyes were assigned as RA (15%) by the Waring classification. Interestingly, 5 of the 15 bilateral cases were found to be inconsistent, among which, four cases were inconsistent with the degree of bilateral, and another one was in a different classification. According to the features of UBM images, cases 11 and 21 were both diagnosed as PA type II in the right eye and PA type I in the left eye; case 15 was diagnosed as PA type II in the right eye and PA type III in the left eye; case 23 was diagnosed as PA type III in the right eye and PA type II in the left eye; and case 26 was diagnosed as PA in the right eye and RA in the left eye.

Previous reports have shown that the penetrating keratoplasty (PKP) had been performed on some PA patients to treat the corneal opacity [14, 25]. In this study, all patients were involved because of CCO and confirmed anterior segment abnormality by UBM. According to our UBM image classification, PA with CCO is classified into three types. From the UBM Dx-Type I to III, the degree of anterior segment abnormality gradually increased. The cause of UBM Dx-Type I corneal opacity is relatively simple, just involving corneal DM and endothelium defect, which is a good indication for PKP. Corneal DM and endothelium defect and iridocorneal adhesion are the most common causes of UBM Dx-Type II. Thus, the iridocorneal adhesion-involved area in different individuals is different. Some patients are not suitable for PKP, while others need PKP combine with anterior segment reconstruction. UBM Dx-Type III is on the fundamental alteration of UBM Dx-Type I or II, with cataract formation, or keratolenticular. A few patients not only could receive PKP but also need to consider combined cataract extraction. UBM Dx-Type IV is consistent with the type of RA, whose corneal opacity is mild. UBM showed severe corneal edema in some patients of UBM Dx-Type IV might be related to poor intraocular pressure (IOP) control, and IOP could be controlled by medication or surgery. When pupillary occlusion is found in UBM, pupilloplasty may be considered. Because of the abnormal structure of anterior chamber and angle, PA and RA often have secondary glaucoma, no matter which of the above treatments, long-term monitoring of intraocular pressure is needed.

## 5. Conclusions

This study indicates that UBM not only could accurately evaluate the anterior segment abnormalities in CCO infants but also would be a step forward for the management of PA- and RA-associated CCO. Moreover, the classification may play a role in guiding treatment recommendations in the future.

## Figures and Tables

**Table 1:** Demography data of the patients with PA- and RA-associated CCO.

Case	Gender	Age (days)	Eyes	Degree of corneal opacity
1	F	7	B	Severe
2	M	790	B	Medium
3	M	120	R	Mild
4	F	26	R	Severe
5	F	113	B	Severe
6	M	7	B	Medium
7	F	57	R	Severe
8	F	44	B	Medium
9	F	150	R	Severe
10	M	910	L	Medium
11	M	22	B	Severe
12	M	180	B	Severe
13	M	1095	L	Mild
14	F	455	B	Medium
15	M	120	B	Medium
16	F	1095	L	Severe
17	M	575	B	R: severe L: mild
18	M	455	R	Severe
19	M	515	B	Mild
20	F	575	R	Medium
21	M	425	B	R: severe L: medium
22	F	395	L	Medium
23	F	300	B	Severe
24	F	240	R	Severe
25	F	270	R	Medium
26	M	21	B	R: severe L: mild

Eyes: B = bilateral, R = right, L = left.

**Table 2:** Findings from UBM.

Case	Eyes	UBM finding	UBM dx-types
1	B	Abnormal strand of peripheral iris extends to the protruding Schwalbe line	IV
2	B	DM and endothelium with discontinuous echo and abnormal filamentous iris extends to the border of the posterior corneal defect	II
3	R	A flat echo consistent with the echo intensity of the iris in the pupil's area and an abnormal strand of peripheral iris extends to the protruding Schwalbe line	IV
4	R	DM and endothelium with heterogenous echo, the bilateral irises adhered to the peripheral cornea, and the lens' echo was enhanced and penetrated into the anterior chamber	III
5	B	DM and endothelium with iridocorneal adhesion and hyperechoic lens penetrated into the anterior chamber	III
6	B	DM and endothelium with discontinuous echo and iridocorneal adhesion	II
7	R	DM and endothelium with discontinuous echo and abnormal filamentous iris extends to the border of the posterior corneal defect; heterogenous hyperechoic lens	III
8	B	DM and endothelium with discontinuous echo	I
9	R	Abnormal strand of iris extends to the distention area of DM and endothelium	II
10	L	The iris adhered to the peripheral cornea and hyperechoic lens	III
11	B	R: DM and endothelium with heterogenous echo and abnormal filamentous iris extends to the border of the posterior corneal defect	R: II
		L: DM and endothelium with heterogenous echo	L: I
12	B	R: DM and endothelium with discontinuous echo, the abnormal bilateral strand of the iris extends to the border of the posterior corneal defect	II
		L: DM and endothelium with iridocorneal adhesion	
13	L	The iris was thickening, a discontinuous echo consistent with the echo intensity of the iris in the pupil's area, and an abnormal strand of peripheral iris extends to the protruding Schwalbe line	IV
14	B	DM and endothelium with heterogenous echo and the lens' echo wre enhanced and penetrated into the anterior chamber, near the posterior surface of the cornea	III
15	B	R: DM and endothelium with discontinuous echo and abnormal filamentous iris extends to the border of the posterior corneal defect	R: II
		L: DM and endothelium with heterogenous echo; the lens' echo was enhanced	L: III
16	L	DM and endothelium with discontinuous echo and abnormal filamentous iris extends to the border of posterior corneal defect	II
17	B	R: DM and endothelium with heterogenous echo, the iris was ante displacement, the lens' echo was enhanced and penetrated into the anterior chamber.	III
		L: DM and endothelium with discontinuous echo, the echo on the anterior surface of the irises was discontinued with iridocorneal adhesion, and the echo of the lens was enhanced	
18	R	DM and endothelium with heterogenous echo and abnormal filamentous iris extends to the border of the posterior corneal defect	II
19	B	DM and endothelium with discontinuous echo and iris thickening	I
20	R	The iris adhered to the peripheral cornea and hyperechoic lens	III
21	B	R: DM and endothelium with discontinuous echo and abnormal filamentous iris extends to the border of the posterior corneal defect	R:II
		L: DM and endothelium with discontinuous echo	L: I
22	L	DM and endothelium with discontinuous echo, iris thickening, and abnormal filamentous iris extends to the border of the posterior corneal defect	II
23	B	R: DM and endothelium with discontinuous echo and abnormal filamentous iris extends to the border of the posterior corneal defect and hyperechoic lens	R:III
		L: DM and endothelium with discontinuous echo and abnormal filamentous iris extends to the border of the posterior corneal defect	L: II
24	R	DM and endothelium with heterogenous echo, the iris was antedisplacement, and iridocorneal adhesion with the central cornea	II
25	R	Iris thinning and abnormal strand of peripheral iris extends to the protruding Schwalbe line.	IV
26	B	R: DM and endothelium with discontinuous echo and an abnormal filamentous iris extends to the border of the posterior corneal defect	R: II
		L: the iris was antedisplacement, an abnormal strand of peripheral iris extends to the protruding Schwalbe line, and pupil disappears	L: IV

Eyes: B = bilateral, R = right, L = left

**Table 3:** UBM imaging features classification.

Type	Eyes	%	UBM imaging features
UBM Dx-type I	6	15.0	DM and endothelium with heterogenous or discontinuous echo, with corneal stroma echo-enhanced or shallow anterior chamber
UBM Dx-type II	16	40.0	On the basis of UBM Dx-type I, with an abnormal strand of iris extending to the border of posterior corneal defect or with iridocorneal adhesion
UBM Dx-type III	12	30.0	On the basis of UBM Dx-type I or II, with an abnormal hyperechoic lens, lens luxation, or keratolenticular adhesion
UBM Dx-type IV	6	15.0	The echoes of the DM and the endothelium were continuous, with corneal stroma echo-enhanced, an abnormal strand of peripheral iris extends to the protruding Schwalbe line, often accompanied by iris stroma or pupil heteromorphism, with a shallow or flat anterior chamber
Total	40	100.0	

**Table 4:** UBM feature of PA and RA consistent with the Warning GO's classification.

Types	Posterior corneal defect	Posterior corneal defect with iridocorneal adhesion	Keratolenticular adhesion or cataract formation	Ring-shaped Schwalbe line	Strand of peripheral iris extending to the protruding Schwalbe line	Iris stroma or pupil heteromorphism
UBM Dx-type I	√					
UBM Dx-type II	√	√				
Peters' anomaly I	√	√				
UBM Dx-type III	√	√	√			
Peters' anomaly II	√	√	√			
UBM Dx-type IV				√	√	√
Rieger's anomaly				√	√	√

## Data Availability

The data sets generated and analyzed during the present study are available from the corresponding author on reasonable request.
